# Archaea dominate the microbial community in an ecosystem with low-to-moderate temperature and extreme acidity

**DOI:** 10.1186/s40168-019-0623-8

**Published:** 2019-01-28

**Authors:** Aleksei A. Korzhenkov, Stepan V. Toshchakov, Rafael Bargiela, Huw Gibbard, Manuel Ferrer, Alina V. Teplyuk, David L. Jones, Ilya V. Kublanov, Peter N. Golyshin, Olga V. Golyshina

**Affiliations:** 1 National Research Center “Kurchatov Institute”, Akademika Kurchatova sq., 1, Moscow, 123182 Russia; 2 Winogradsky Institute of Microbiology, Federal Research Center for Biotechnology, Russian Academy of Sciences, Prospect 60-Letiya Oktyabrya 7/2, Moscow, 117312 Russia; 3 School of Natural Sciences, Bangor University, Deiniol Rd, Bangor, LL57 2UW UK; 4 Institute of Catalysis CSIC, 28049 Madrid, Spain; 5 UWA School of Agriculture and Environment, University of Western Australia, Crawley, WA 6009 Australia; 6 Centre for Environmental Biotechnology, Bangor University, Deiniol Rd, Bangor, LL57 2UW UK

**Keywords:** *Cuniculiplasma*, *Cuniculiplasmataceae*, *Thermoplasmatales*, Acidophilic archaea, Terrestrial miscellaneous Euryarchaeotal group (TMEG), Acid mine drainage (AMD) systems, Mine-impacted environments

## Abstract

**Background:**

The current view suggests that in low-temperature acidic environments, archaea are significantly less abundant than bacteria. Thus, this study of the microbiome of Parys Mountain (Anglesey, UK) sheds light on the generality of this current assumption. Parys Mountain is a historically important copper mine and its acid mine drainage (AMD) water streams are characterised by constant moderate temperatures (8–18 °C), extremely low pH (1.7) and high concentrations of soluble iron and other metal cations.

**Results:**

Metagenomic and SSU rRNA amplicon sequencing of DNA from Parys Mountain revealed a significant proportion of archaea affiliated with *Euryarchaeota,* which accounted for ca. 67% of the community. Within this phylum, potentially new clades of *Thermoplasmata* were overrepresented (58%), with the most predominant group being “E-plasma”, alongside low-abundant *Cuniculiplasmataceae*, ‘Ca. Micrarchaeota’ and ‘Terrestrial Miscellaneous Euryarchaeal Group’ (TMEG) archaea, which were phylogenetically close to *Methanomassilicoccales* and clustered with counterparts from acidic/moderately acidic settings. In the sediment, archaea and *Thermoplasmata* contributed the highest numbers in V3-V4 amplicon reads, in contrast with the water body community, where *Proteobacteria*, *Nitrospirae*, *Acidobacteria* and *Actinobacteria* outnumbered archaea. Cultivation efforts revealed the abundance of archaeal sequences closely related to *Cuniculiplasma divulgatum* in an enrichment culture established from the filterable fraction of the water sample. Enrichment cultures with unfiltered samples showed the presence of *Ferrimicrobium acidiphilum*, *C*. *divulgatum*, ‘*Ca*. Mancarchaeum acidiphilum Mia14’, ‘*Ca*. Micrarchaeota’-related and diverse minor (< 2%) bacterial metagenomic reads.

**Conclusion:**

Contrary to expectation, our study showed a high abundance of archaea in this extremely acidic mine-impacted environment. Further, archaeal populations were dominated by one particular group, suggesting that they are functionally important. The prevalence of archaea over bacteria in these microbiomes and their spatial distribution patterns represents a novel and important advance in our understanding of acidophile ecology. We also demonstrated a procedure for the specific enrichment of cell wall-deficient members of the archaeal component of this community, although the large fraction of archaeal taxa remained unculturable. Lastly, we identified a separate clustering of globally occurring acidophilic members of TMEG that collectively belong to a distinct order within *Thermoplasmata* with yet unclear functional roles in the ecosystem.

**Electronic supplementary material:**

The online version of this article (10.1186/s40168-019-0623-8) contains supplementary material, which is available to authorized users.

## Background

Prokaryotic organisms which optimally grow at pH 3 and below are termed acidophiles. Acidophilia is a trait widely distributed among numerous prokaryotic phyla. Historically, a few bacterial species, such as *Acidithiobacillus* spp. and *Leptospirillum* spp. were believed to be the most ubiquitous and abundant acidophiles. However, current knowledge suggests that acidophilic bacterial diversity stretches over numerous representatives of *Proteobacteria*, *Nitrospirae*, *Firmicutes*, *Actinobacteria*, *Acidobacteria*, *Aquificae* and *Verrucomicrobia* [1, 2]. Furthermore, the signatures of *Chloroflexi* and various uncultured candidate taxa have been documented in acidic environments [3]. In these environments, culture-independent molecular techniques suggested the affiliation of the archaeal acidophilic component to the phyla *Euryarchaeota*, *Crenarchaeota*, *Thaumarchaeota*, ‘*Candidatus* Parvarchaeota’ and “*Ca*. Micrarchaeota” [1], whereas the few well-studied archaeal acidophilic isolates exclusively belonged to the order *Thermoplasmatales* of *Euryarchaeota* and to the phylum *Crenarchaeota* [2, 4, 5]. More recent metagenomic surveys of acidic sites have, however, revealed that a large proportion of organisms therein are affiliated with yet uncultured and unstudied taxa within *Thermoplasmata* [1, 6, 7]. Groups of uncultured archaea affiliated with the order *Thermoplasmatales* [8] have been predicted in acid mine drainage (AMD), one of which, ‘G-plasma’, has successfully been isolated, characterised and described as *Cuniculiplasma divulgatum* [9, 10]. A handful of members from the families *Ferroplasmaceae* and *Cuniculiplasmataceae* are currently the only isolated and characterised mesophilic extremely acidophilic species of archaea. The major physico-chemical drivers regulating the relative abundances of the bacterial and archaeal components in the acidophilic microbiome, however, remain unclear. Temperature, pH, redox potential and dissolved oxygen are considered the main factors influencing the composition of any acidic microbial community [11]. Temperature is often assumed to be the most important factor regulating the relative abundance of archaeal vs bacterial components with a general assumption that bacteria are more prevalent at lower temperatures [12]. In contrast, the prevailing pH is considered to be of secondary importance [12]. It has been shown that at pH < 2, the relative abundance of archaea in AMD systems increases with up to 25% of all prokaryotes belonging to this Domain [2]. It was also reported that at pH values below 1.9, the relative abundance of *Thermoplasmatales* archaea increased at the expense of populations of proteobacteria [13]. Moreover, biofilm samples in sites with pH values of about 1 and temperatures around 38 °C taken from underground site on Richmond Mine (CA, USA) demonstrated an extremely high proportion of archaeal reads (69–98% of the total) [14]. Representatives of *Euryarchaeota* and ‘*Ca*. Parvarchaeota’ were reported as major components in certain acidic environments, characterised by pH 1.9 and 43.3 °C and by pH 2.7 and 21.4 °C, respectively [15]. Furthermore, the data of [16, 17] pointed at the prevalence of archaeal reads in low pH ecosystems in warm subtropical climate with an annual mean temperature of 18 °C. However, the Archaea inhabiting ecosystems with rather moderate or constantly low temperatures are frequently considered to constitute only a minor proportion of the entire microbial community or to be completely absent [3, 11, 18]. Parys Mountain (Parys Mt), located on the Island of Anglesey (North Wales, UK), is an example of such a cool environment, and the microbiome of its acidic stream was chosen for the present study.

Parys Mt is historically one of the most important copper mines in Europe and is a site of intense geological interest: there is evidence for ancient black smokers, which existed 480–360 M years BP at the site of Parys Mt [19]. During a volcanic eruption at the end of Silurian, sulfidic minerals composed mostly of copper, but also iron, lead and zinc formed large ore deposits [20]. These ores were mined for copper from the early Bronze Age until 1900 when all commercial mining ceased. Currently, the site is characterised by open casts with an underground system of AMD streams and man-made channels. Significant research has previously been conducted to investigate microorganisms and their communities associated with this site, which has resulted in a number of new bacterial species being isolated and described and a few PCR-based studies using terminal restriction fragment length polymorphism (T-RFLP) fingerprinting and 16S rRNA gene amplicon libraries [18, 21, 22]. Only a minor proportion of archaeal signatures affiliated with *Euryarchaeota* were detected in low pH subterranean water and in acidic streamer of Dyffryn Adda on Parys Mt, characterised by 8–9 °C in subsurface environments and air temperatures fluctuating on average between 8 and 18 °C across seasons [18, 21].

The primary goal of the present study was the analysis of prokaryotic community composition in an acidic stream, a shallow mine water flow, which originates from an open pit pond passing through the sulfidic deposits/gravel of Parys Mt. This system is different to the filamentous growth/acid streamer ecosystem within the channel Dyffryn Adda studied previously [18] but represents a free running acidic mine water flow without a characteristic red colour but with some algal growth on the surface (Additional file 1: Figure S1). Another goal of this study was attempted to cultivate archaea using sample pre-treatment and amendment with complex organic substrates to establish the taxonomic affiliation of culturable archaea.

## Results and discussion

### Chemical composition of sediment and water samples

Chemical analysis was performed on two types of samples, the sediment and the overlying water (Table 1, Additional file 1: Table S1). Overall, both contained high contents of iron and sulphur, respectively ca. 66.7 and 217 g/kg in sediment and 1.93 and 80.8 g/L in water sample. Al, K, Pb and Ti in the sediment and Zn, Cu, Pb and Ca for water fraction were also present in high concentrations. Total C and N values in the sediments were detected at 2.8% and 0.3% (*w*/*w*), respectively. In situ measurements for pH indicated 1.74 for both sample types. Upon arrival to laboratory, the values of pH showed a slight increase to 2.84 for the sediment and 1.90 for the aqueous fraction. The redox measured in the laboratory was found to be + 272 mV in the sediment and + 577 mV in the water sample. Electrical conductivity was found to be very high in the water sample (10.3 mS/cm). Total chemical data are shown for the particulate fraction in Table 1 and for the water fraction in Additional file 1: Table S1.

**Table 1:** Chemical data obtained in the samples of sediment and water of the acidic stream of Parys Mt. Values represent means ± SEM

Parameter	Unit	Sediment	Water
Electroconductivity	(mS/cm)	7.41 ± 0.66	10.26 ± 0.09
pH		2.75 ± 0.14	1.90 ± 0.05
Redox	(mV)	272 ± 38	577
Total Al	(mg/kg)	12,375 ± 2092	< 1
Total S	(mg/kg)	1932 ± 543	80,754 ± 2260
Total K	(mg/kg)	8966 ± 1146	204 ± 33
Total Ca	(mg/kg)	574 ± 189	1112 ± 38
Total Ti	(mg/kg)	1642 ± 151	< 1
Total V	(mg/kg)	245 ± 34	< 1
Total Cr	(mg/kg)	309 ± 71	660 ± 74
Total Mn	(mg/kg)	191 ± 58	< 1
Total Fe	(g/kg)	66.7 ± 10.6	216.5 ± 5.2
Total Ni	(mg/kg)	11 ± 1	< 1
Total Cu	(mg/kg)	495 ± 108	8477 ± 213
Total Zn	(mg/kg)	632 ± 102	10,041 ± 294
Total As	(mg/kg)	828 ± 51	244 ± 23
Total Sr	(mg/kg)	38 ± 5	51 ± 2
Total Pb	(mg/kg)	4122 ± 1111	1491 ± 129
Stone content	(%)	5.4 ± 2.2	
Dry bulk density	(g/cm^3^)	0.87 ± 0.16	
Water filled porosity	(%)	58.7 ± 0.9	
Air filled porosity	(%)	8.58 ± 5.47	
Total C	(%)	2.8	
Total N	(%)	0.30	
C:N ratio		9.9	

### Microbial community composition in sediment and water samples

**Fig. 1:**
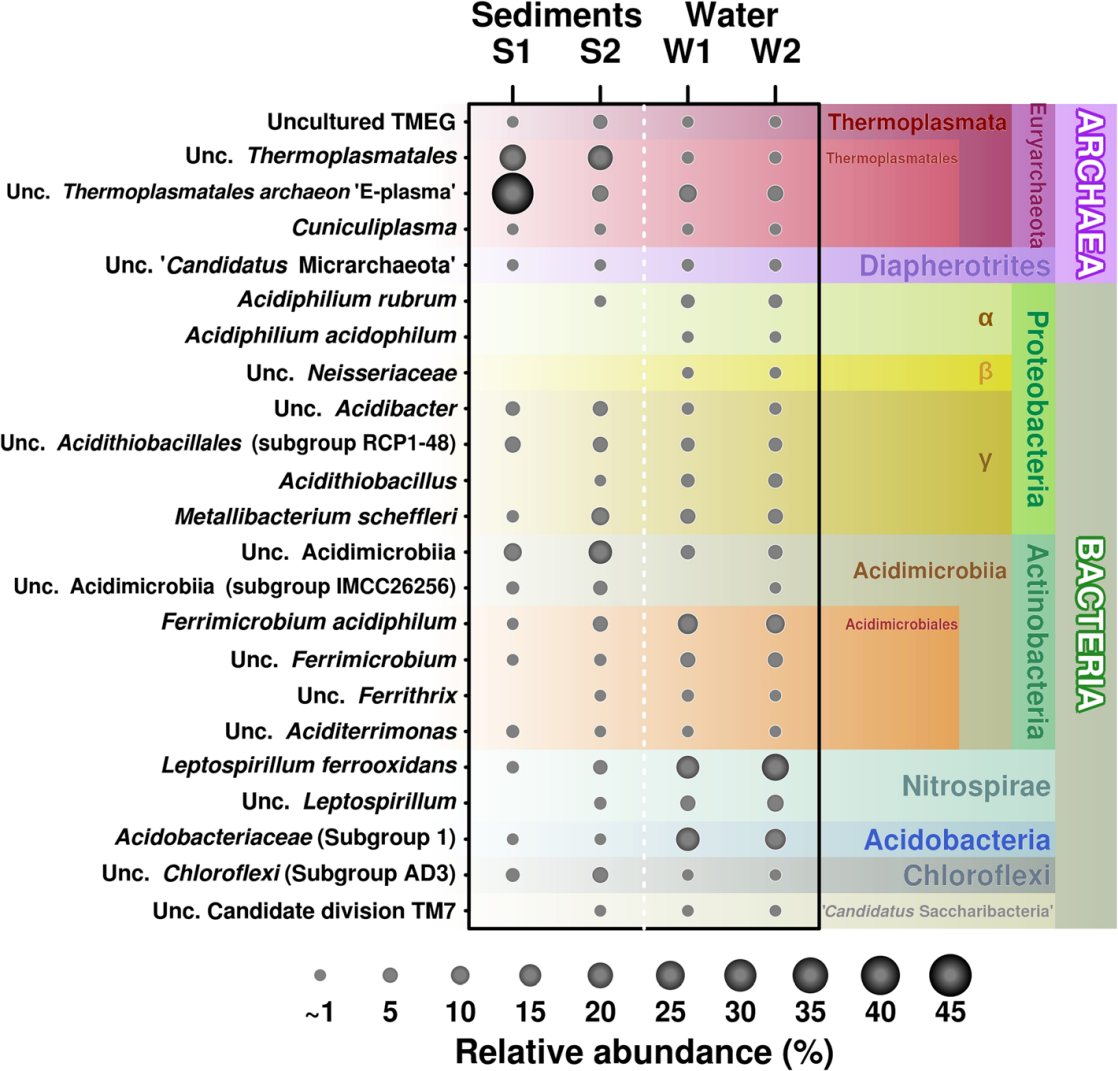
Abundance of SSU rRNA barcoded amplicon reads in water and sediment samples of acidic stream. Figure was performed under R programing environment using basic R packages

Analysis of V3-V4 rRNA region profiling data revealed 123 significantly presented ASVs, corresponding to 12 different phyla (Fig. 1). Analysis of microbial diversity in the samples showed that while the water communities were similar between replicates, the sediment was much more heterogeneous: both alpha-diversity measures in the two sediment samples were significantly different from each other (Additional file 1: Figure S2). This is a similar finding to samples taken from the Los Rueldos mine [7], and represents the physical non-uniformity of the sediment.

#### Sediment-associated archaea

In both analysed sediment samples, euryarchaeal class *Thermoplasmata* was the most abundant group constituting 31.7–65.5% of all amplicon reads. Within class *Thermoplasmata*, the *Thermoplasmatales* representatives were present in the highest abundance, with a dominance of ‘E-plasma’ reads (up to 43.5%, MH574490). The second abundant archaeon (ASV MH574492) (5.9–17.2% of total reads) was similar to the uncultured *Thermoplasmatales* archaeon ‘B_DKE’, previously reported in stable acidophilic enrichment of samples from a pyrite mine in the Harz Mountains, Germany [23]. The uncultured member of *Thermoplasmatales* (ASV MH574497), which is common in extreme acidophilic sites, was also represented constituting between 3.4 and 12.0% of total reads. The members of the latter group were detected in the abandoned mercury mine in Los Rueldos [7], those are almost equally related to *Thermoplasmatales* archaeon ‘A-plasma’ (94% 16S rRNA gene sequence identity) and *Cuniculiplasma divulgatum* (93% identity). Another novel *Thermoplasmatales* archaeon was detected in minor quantities (< 1.7% reads) and was represented by ASV MH57451 and is phylogenetically positioned between ‘A-plasma’ and ‘E-plasma’ (both 94% identity). Archaeal *Cuniculiplasma-*related taxa with cultivated representatives, earlier been isolated from this same mine site [10] were found only in minor quantities (0.2–0.5%). Small numbers of reads were also apparent for TMEG (0.2–4.5%) and for ‘*Ca*. Micrarchaeota’ (0.3–0.4%), which resembles the earlier reports on the low TMEG numbers in Los Rueldos acidic ecosystem [1].

#### Planktonic archaea

In surface water samples, archaea constituted a much lower proportion in comparison to sediment (8.1–11.7% of the total reads). ‘E-plasma’ and ‘B_DKE’ were also detected in moderate numbers (6.4–9.4% and 0.8–1.6%, respectively). *Cuniculiplasma* and TMEG-related reads were only present in very small quantities (0.7–0.3% and 0.2–0.08%, correspondingly) as was ‘*Ca*. Micrarchaeota’ (1.7–1.4%). In relation to this, an increase of the content of ‘*Ca*. Micrarchaeota’ organisms in water was seen, albeit their overall total representation was below 2%. The member of ‘*Ca*. Micrarchaeota’, namely ‘*Ca*. Mancarchaeum acidiphilum’ originated from this environment was reported recently to be co-cultured in vitro with *Cuniculiplasma divulgatum* [24].

#### Sediment-associated bacteria

Barcoded V3-V4 amplicon sequencing analysis revealed that sediment-associated groups encountered for 14.9–29.9% reads derived from *Actinobacteria* and 12.3–21.8% from *Proteobacteria*, the largest proportion of which was attributed to yet uncultured organisms. Other groups were found in lower quantities. *Actinobacteria* can constitute a large proportion of organisms in nutrient-limited environments [25]. Thus, actinobacterial species *Ferrimicrobium acidiphilum* was isolated from the mine site in North Wales and was described as a mesophilic heterotrophic iron oxidiser/reducer and as the predominant bacteria in Parys Mt AMD systems [18, 26]. The phylum *Proteobacteria* is known to include the most common acidophilic bacteria [1] accounted for 12.3–21.8% reads affiliated with γ-*Proteobacteria* Subgroup 1 (2.4–4.6%) and *Metallibacterium* spp. (family *Xanthomonodaceae*) (1.1–9.4%) detected alongside unclassified proteobacterial sequences, followed by *Acidithiobacillales ‘*cluster RCP 1-48’ (5.7–6.3%). *Metallibacterium schleffleri*, the cultured representative of metallibacteria, was described as acid-tolerant and organotrophic bacterium, sequences of related organisms were found to be broadly represented in mine-impacted environments being found in both biofilms and sediments [27]. *Acidithiobacillales* ‘cluster RCP 1-48’ was previously found to be associated with anaerobic sediments of Rio Tinto and mercury mine streamers [7, 28]. Furthermore, the reads from candidate phylum AD3 (3.6–6.2%) and *Nitrospiraceae* (1.6–5.8%) were also identified. Additionally, a few cyanobacterial signatures were also determined in the sediments.

#### Planktonic bacteria

The principal bacterial members inhabiting the water fraction were *Leptospirillum* spp. (*Nitrospirae*) (22.9–30.6% of total reads), that are renowned iron oxidising colonisers of acidic environments and are considered as the one of the most low pH-resistant bacterial species [1]. These were followed by *Ferrimicrobium* spp. (*Actinobacteria*) (11–12.2*%)* and other *Acidimicrobiia* from the same phylum*.* These patterns have previously been detected and reflect the predominance of *Leptospirillum* in mine waters and of *Actinobacteria* in filamentous growth matrix [18]. Similarly, the elevated presence of *Thermoplasmata* has been observed in sediment columns of Rio Tinto with *Leptospirillum* prevailing in the planktonic biomass [29].

Other important groups were *Proteobacteria* (19.6–22.3%) with detectable number of reads related to γ-proteobacterial genus *Metallibacterium* (5.9–6%), *Acidithiobacillus* (family *Acidithiobacillaceae*) (3.4–4.6%) and α-proteobacterial *Acidiphilium* (family *Acetobacteraceae*) (3.6–3.9%). Above taxa contain a number of well-studied organisms. Thus, chemolitotrophic, mesophilic, moderately thermophilic and low temperature-adapted *Acidithiobacillus* spp. were the first isolated acidophiles and possibly the best-studied species from acidic environments [1]; heterotrophic iron-reducing *Acidiphilium* are also known to be widely represented in low pH settings [1]; significant quantities of *Acidobacteria* (13.2–17.3%) were also revealed, mainly affiliated to a Subdivision 1 cluster. It was reported that the highest relative abundance of subdivision 1 cluster of *Acidobacteria* was present in ecosystems with moderately acidic pH (about 4), with the only acidobacterial species known to inhabit AMD environments being the mesophilic and obligately heterotrophic *Acidobacterium capsulatum* [1, 30]. Other bacterial taxa were present in only minor quantities (< 2% of the total). Interestingly, *Actinobacteria*, whose cultured representatives were shown to be heterotrophic, were among the most abundant bacterial counterparts in both fractions. Other groups of bacteria *Acidithiobacillales*, *Xanthomonodales* (*Proteobacteria*) and *Chloroflexi* were present in larger proportions in sediment as compared to acidic water fractions (Fig. 1).

### Metagenomic sequencing of AMD microbiome

**Fig. 2:**
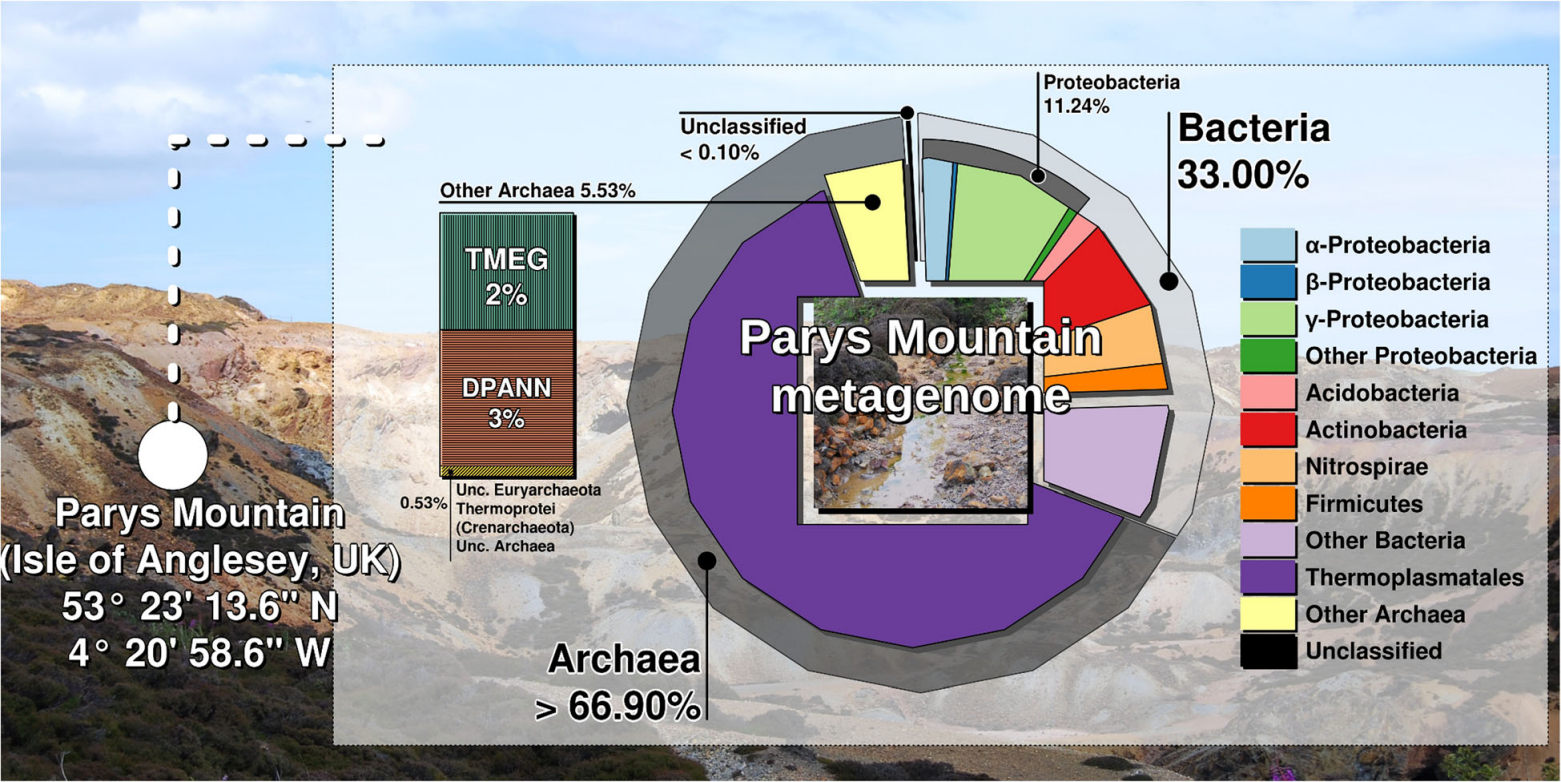
Taxonomic classification of microbial community from metagenomic DNA sequencing data. Taxonomic classification was obtained using ^1^GraftM, classifying short reads belonging to marker genes from metagenomic datasets. Graphical representation was performed under R programing environment using basic R packages. https://github.com/geronimp/graftM (This program does not have a publication yet, but they suggest to cite their github page)

**Fig. 3:**
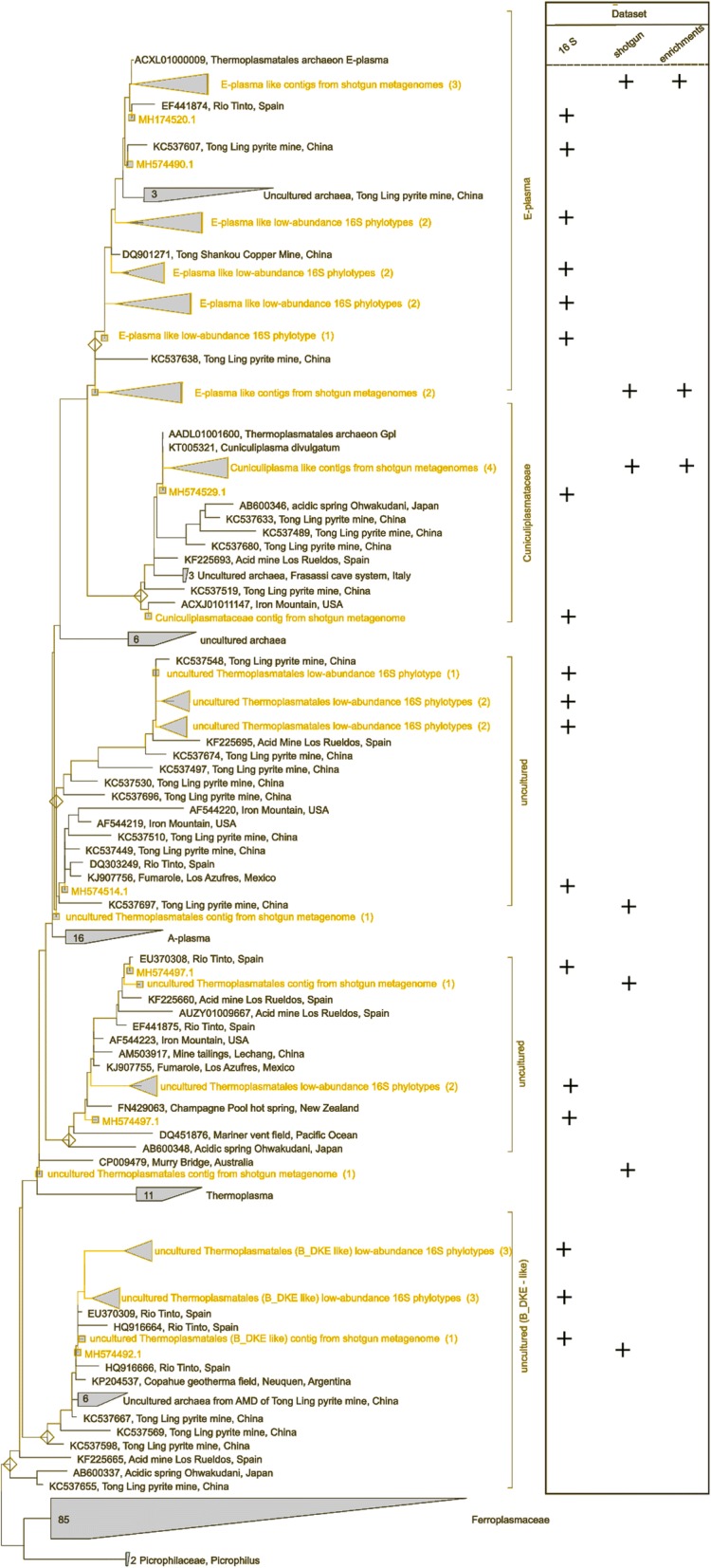
16S rRNA gene sequence-based phylogenetic reconstruction of *Thermoplasmatales* clones from Parys Mt samples obtained in the course of this work. V3-V4 hypervariable region amplicon sequences and sequences extracted from metagenome contigs are highlighted by green. Number of amplified sequence variants, corresponding to each branch indicated in brackets. Reference sequences (brown) were taken from the Silva SSU 132 Ref NR 99 database (see the methods section). ‘Plus’ sign denotes presence of corresponding sequences in 16S V3-V4 amplicon, environmental and enrichment metagenomes

Metagenomic analysis of water interface and sediment samples revealed that the microbial community consisted of Archaea (67%) and Bacteria (33%) (Fig. 2, Additional file 1: Figure S3). Among the archaea, *Euryarchaeota* accounted for 64%, ‘*Ca*. Micrarchaeota’ for 3% and ‘*Ca*. Parvarchaeota’ for 0.2%, both belonging to the candidate superphylum ‘DPANN’ (‘*Diaphetotrites*-*Parvarchaeota*-*Aenigmarchaeota*-*Nanohaloarchaeota*-*Nanoarchaeota*’), with the rest of the archaea contributing 0.5% of total prokaryotic reads. *Thermoplasmata* (64% from all prokaryotes) were the most abundant within euryarchaea, the majority of the former was represented by *Thermoplasmatales* (62%), specifically by unclassified *Thermoplasmatales* (58% of total sequencing reads) and *Cuniculiplasmataceae* (4%). Unclassified *Thermoplasmatales* were affiliated mainly with ‘E-plasma’ clonal variant. A better understanding the physiology and in situ function of this group could be achieved after its successful cultivation. Phylogenetic positioning of archaeal sequences deduced by V3-V4 amplicon sequencing and metagenome shotgun sequencing is presented in Fig. 3. The indigenous *Thermoplasmatales* archaea showed high SSU rRNA gene sequence identities with counterparts from other acidic environments: Iron Mountain [8], Tongling AMD samples (see Accession Numbers in Fig. 3), floating macroscopic filaments from Rio Tinto [28, 31], Pb/Zn mine tailing [32] and Frasassi cave system/acidic snottite biofilm [33]. All these *Thermoplasmatales*-related sequences belong to organisms still evading isolation. Since the archaea of the order *Thermoplasmatales* are known scavengers of proteinaceous substrates from microbial biomass [5], we hypothesise that the metabolic functioning Parys Mt archaea may be considered as heterotrophic to a large extent. The substantial archaeal proportions in samples with low total C and N values (Table 1) and lesser numbers of prokaryotic primary producers (*Leptospirillum* sp. or *Acidithiobacillus* spp.) may be associated with green algae populating the surface (*Chlamydomonas* spp.). Accordingly, *Chlamydomonas* is a renowned inhabitant of AMD systems [34] and is capable of exudation of low molecular weight organic compounds [35] that can further be utilised by other members of the microbial consortia. Altogether, these archaea, due to their elevated numbers, can be considered as significant contributors to the community functioning and to elemental cycling (carbon and probably iron) in this low-temperature ecosystem. Another factor favouring the elevated numbers of *Thermoplasmatales* is the high acidity of this environment. Preliminary analysis of the Parys Mt. microbial community showed that a large number (almost 57%) of total Illumina reads, resulting from shotgun sequencing of Parys Mt metagenome, were related to *Thermoplasmatales* archaea [24].

Bacteria (33% of the total prokaryotic reads) showed affiliation to *Proteobacteria* (11%), with *γ-Proteobacteria* (8%) and *α-Proteobacteria* (2%) being the major classes. The closest γ-proteobacterial reads were similar to *Xanthomonodaceae*, with a significant number of *Lysobacter* spp. (2% of all prokaryotic and 7% of all bacterial reads). These microorganisms inhabit neutral soil and freshwater environments and are known to be a rich source of antibiotics [36]. *α*-Proteobacterial sequences (2% from total reads) were affiliated with *Rhodospirillales* with other groups present in minor numbers. Representatives of *Rhodospirillales* (*Acidiphilium* spp., *Acidicaldus organivorans*, *Acidocella* spp. and *Acidisphaera* spp.) are known acidophilic heterotrophic bacteria able to use exudates and lysates from primary producers and were detected previously at Parys Mt site Dyffryn Adda streamers [18]. Other bacterial phyla were identified as *Actinobacteria* (6%), *Nitrospirae* (4%), *Bacteroidetes* (3%), *Acidobacteria* (2%), *Firmicutes* (2%) and others, present in minor proportions. *Acidithiobacillales* were represented in lesser quantities (0.3%). Unassigned bacterial sequences comprised 13% of total bacterial reads. The Shannon and Simpson diversity indices were calculated as 1.97 and 0.64, respectively (Additional file 1: Figure S3).

Earlier studies of Parys Mt sites using PCR-based methods revealed the dominance of bacteria with only a minor presence of archaea [22]. In addition, analysis of the Parys Mt Dyffryn Adda AMD community proposed that *Acidithiobacillus ferrivorans* (order *Acidithiobacillales*), ‘*Ferrovum myxofaciens*’ (*Proteobacteria*) and *Acidithrix ferrooxidans* (*Actinobacteria*) were the dominant bacteria in this environment [18]. The ratios of bacteria to archaea have not been assessed in the study; however, archaeal sequences were distantly related to cultured members of *Euryarchaeota* and were much less diverse than bacteria. In addition, the authors concluded that the presence of archaea in other North Wales acidic mine sites is low [18]. In that context, our results found archaea to be in the clear majority. We suggest that this disparity between different studies may be connected with general limitation of PCR-based methods used and the different physico-chemical conditions in both sites, including higher pH values in Dyffryn Adda streamers (2.5 vs 1.8), notable increase in Eh (+ 669 vs + 577 and + 272) and significantly low concentrations of metals [18].

### TMEG and *Methanomassilicoccales*-related archaea

**Fig. 4:**
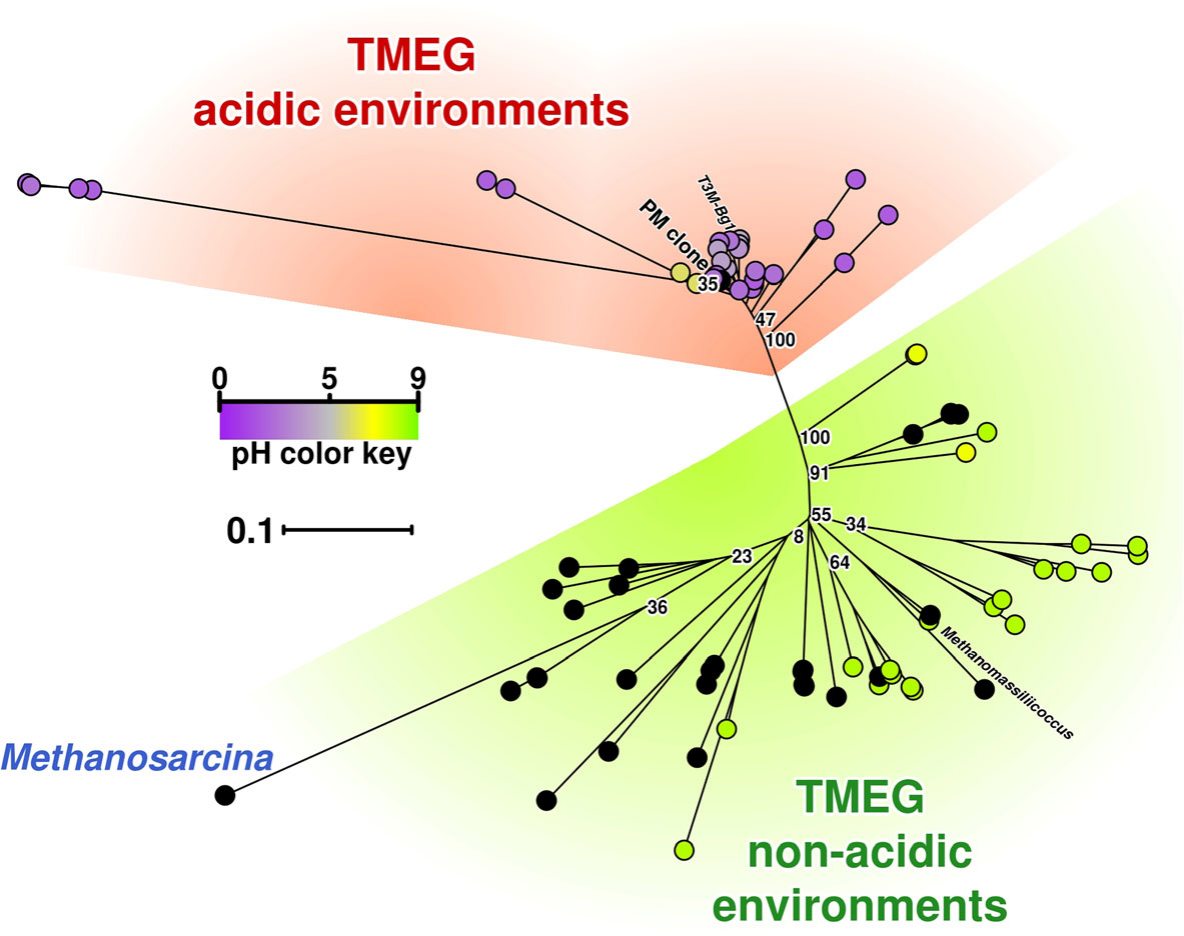
16S rRNA gene sequence-based phylogenetic relationships of microorganisms from the Terrestrial Miscellaneous Euryarchaeotal Group (TMEG). The tree was constructed using a total of 82 16S rRNA sequences that were assigned and documented in the literature to TMEG, including the clone described in this research (referred as *PM clone*) and two sequences from *Methanosarcina* sp. and *Methanomassiliicoccus luminyensis*, respectively. Environmental pH is shown by coloured dots from acidic (purple) to neutral-low basic (green). Sequences from environments with unknown pH are shown in black. Two distinct clusters of sequences can be identified, corresponding to sequences from acidic (red cluster) and non-acidic environments (green cluster). The list of analysed sequences is presented in the Additional file 2: Table S2 ‘TMEG Data’

Additionally, 2% of the sequences were identified as TMEG-related (*Thermoplasmata*). The phylogenetic position of TMEG-related sequences suggested this group had *Methanomassiliicoccus luminyensis* strain B10 as the nearest cultured representative with the 16S rRNA gene sequence identity of 83% (Fig. 4, Additional file 2: Table S2). The phylogenetic tree (Fig. 4) clearly shows clustering of Parys Mt TMEG-related reads with other clones identified in acidic and moderately acidic environments discriminative to sequences from other places.

These archaea are considered to be minor constituents of the communities, but were shown to be more associated with sediment rather than with the water fraction in this particular environment. Recent metagenomic studies indicated that this group might be linked to anoxic sulfate- and methane-containing settings. For instance, it was revealed that the highest number of clones associated with TMEG was detected in sediments with low amounts of SO_4_^2−^ (2 mM) and moderate concentrations of CH_4_ (3.5 mM) [37]. Another study [38] reported on the presence of TMEG in methane hydrate-bearing sediments of freshwater Baikal Lake comprising ca. 15% of the total microbial community in an upper level and at about 10% in sediments. Interestingly, the athalassohaline inland aquatic system, Salar de Huasco, located in the Chilean Altiplano and characterised by low temperatures showed a predominance of this group in a water sample with the highest sulfate concentration (40 mM) [39]. Thus, the physiology and functions of TMEG group still remain completely unexplored either in aquatic environments or sediments [40]. It was predicted that they have a potential to undertake fatty acid oxidation and anaerobic respiration via reduction of sulphite and/or sulfonate (bin Bg1) [41]. In relation to TMEG, an important point was recently made about a strong phylogenetic separation of gut-associated and free-living clades of *Methanomassilicoccales*-related archaea [42]. Furthermore, it was noted that the latter ‘environmental clade’ of *Methanomassilicoccales* was previously assigned to TMEG [43]. We detected the presence of 16S rRNA gene sequences with the identity of about 81–83% to those of *M*. *luminyensis* in sediments of the stream of Parys Mt in barcoded 16S rRNA gene amplicon sequences (V3-V4 region) and by shotgun metagenomic sequencing. Almost identical to Parys Mt sediment, TMEG-derived SSU rRNA gene sequences were earlier detected in La Zarza, Perrunal acid mine effluent (Iberian Pyritic Belt, Spain, HM745465 [44], Rio-Tinto, DQ303248 [31], terrestrial acidic spring AB600345 [45] and many other acidic or slightly acidic environments [7, 46, 47]). In some cases, the affiliation of these sequences to the order *Thermoplasmatales* has been erroneously considered. Our analysis pointed at a compact co-clustering of TMEG-related sequences from a wide range of acidic and moderately acidic ecosystems that were very distinct from the cluster of their counterparts from neutral environments. These acidophilic/acidotolerant TMEG archaea form a group on the level of a new order within *Thermoplasmata*, and are only distantly related to *M*. *luminyensis*. T-RFLP and clone library analyses conducted earlier identified similar organisms in other Parys Mt settings: in impounded water (corresponding microorganisms were referred as a ‘methanogens’) [21] and in filamentous growth streamers in the Dyffryn Adda mine adit [18]. The presence of a group of rRNA gene sequences with about 80% identity to *M*. *luminyensis* was also mentioned in a study of arsenic-rich creek sediments of Carnoulès Mine, France [48], in Los Rueldos acidic streamers ( [7] and elsewhere).

### Enrichment cultures

**Fig. 5:**
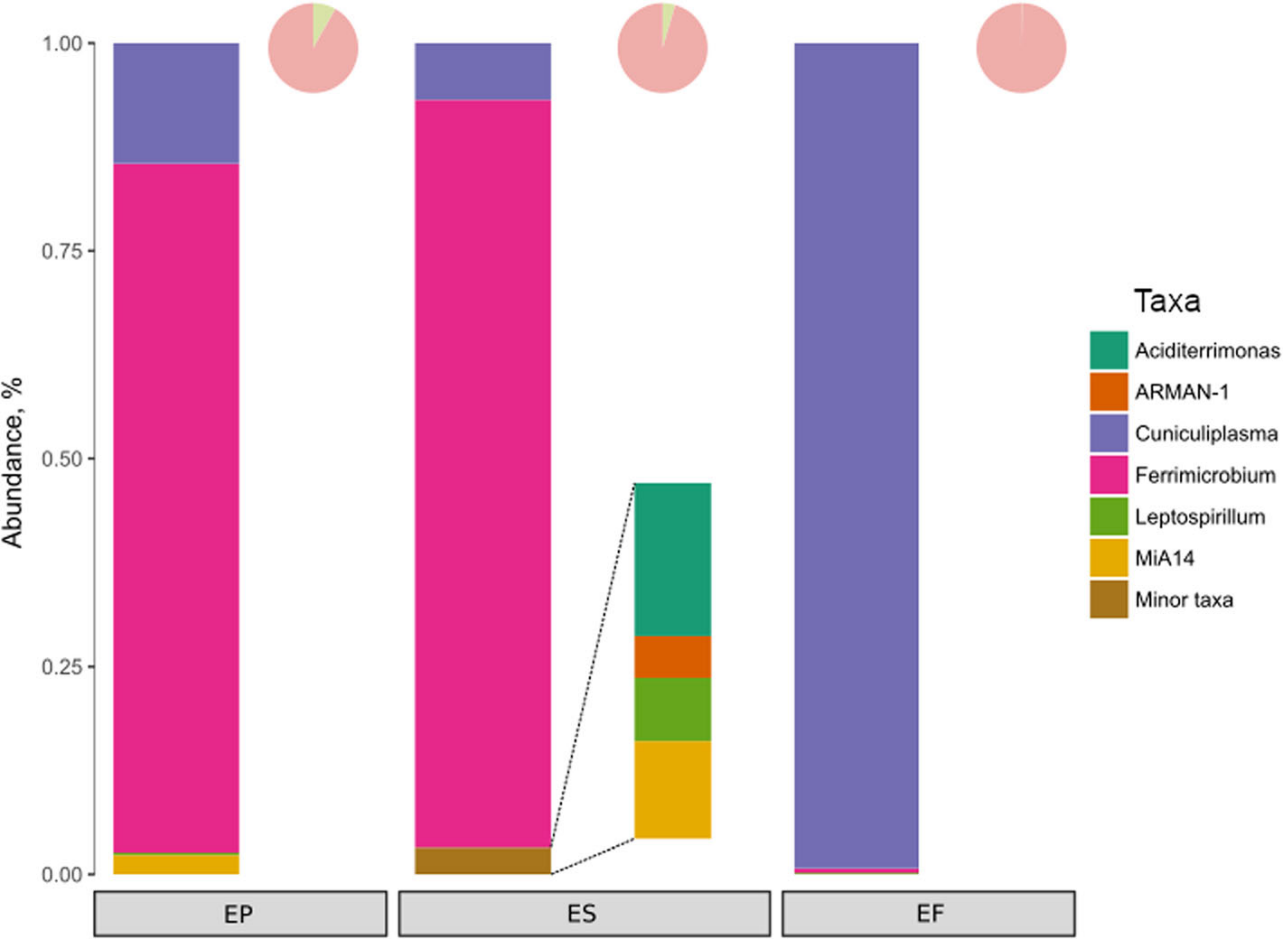
Abundance of archaea and bacteria according to the results of metagenomic sequencing of DNA isolated from enrichment cultures with differential pre-processing methods (samples EP, ES and EF). Proportion of classified (pink) vs. unclassified (light green) reads is shown by small pie-charts on top right corner of each bar

An attempt to enrich and isolate archaea from this environment was undertaken. For this, we applied various treatments of samples, followed by enrichment and consequent shotgun sequencing of these variants (see ‘Methods’ section). After 25 days of cultivation of enriched samples (NN 4 and 5), certain diversification between variants was observed (Fig. 5). The EP culture containing sediment matrix was dominated by *Ferrimicrobium acidiphilum*, showing 99% 16S rRNA nucleotide identity with *Fm*. *acidiphilum* strain T23^T^ previously isolated from the Cae Coch sulphur mine [26]. Archaea were represented by *Cuniculiplasma divulgatum* and ‘*Ca*. Mancarchaeum acidiphilum Mia14’, corresponding to 14.4% and 2.3% of classified metagenomic reads, respectively.

The ES culture was established using the supernatant of the above centrifugate as an inoculum to reduce the numbers of large algal cells and solid particulate matter. Upon incubation, *Fm*. *acidiphilum* was the predominant organism with a lower abundance of *Cuniculiplasmataceae* than in EP culture. Nevertheless, ES culture was significantly more diverse than EP. Small numbers or ‘*Ca*. Micrarchaeota’-related reads, with 16S rRNA gene sequence almost identical to that of ‘A_DKE’ organism from the enrichment culture of ‘Drei Kronen und Ehrt’ pyrite mine microbial consortium [23] and ‘*Ca.* Mancarchaeum acidiphilum’ Mia14, corresponding to 0.4% and 0.9% of classified metagenomic reads, respectively, were detected. Minor bacterial taxa were represented by uncultivated species of *Leptospirillum* (0.6%) and *Aciditerrimonas* (1.3%). Closest 16S rRNA gene sequences for the latter were reported in clones from acid mine effluent of La Zarza, Perrunal mine located in the Iberian Pyritic Belt [44].

Finally, an enrichment culture (EF) was set up using the filtrate which had passed through a 0.45 μm pore membrane. This was used as an inoculum but required a longer cultivation time (45 days). It showed a predominance of *C*. *divulgatum* with only a minor fraction of bacteria (0.7%) of *Leptospirillum* and *Ferrimicrobium* spp*.* In contrast to the other enrichment cultures, no ‘*Ca*. Micrarchaeota’ signatures were detected in the EF culture. However, one needs to consider that similar culturing conditions have been previously optimised for *Cuniculiplasma divulgatum* [9] and may be less favourable to other Parys Mt archaea.

## Conclusions

Our metagenomic and metabarcoding amplicon sequencing analyses have unambiguously pointed at the predominance of archaea in the low-to-moderate temperature environment of Parys Mt, in contrast to earlier considerations of the prevalence of bacteria in such environments. One particular group of uncultured *Thermoplasmatales*, dubbed ‘E-plasma’ was present in a clear majority. The overwhelming archaeal numbers in this natural acidic microbiome implies their pivotal functional and ecological role in the community. Our sample pre-treatment and cultivation attempts were successful in obtaining enrichment cultures with very high proportion of *Cuniculiplasma* spp., which, however were represented in natural samples as a minor component. An important finding is related to the TMEG archaeal group previously overlooked in acidophilic communities. Our analysis showed their presence in the microbial community of Parys Mt and suggested that at a global scale, there is a strong phylogenetic clustering of TMEG sequences from the low pH settings that form a separate clade at the level of the Order within *Thermoplasmata*. Considering the majority of microbiome constituents were represented by yet uncultured organisms, this environment is a good resource for isolation of apparent new archaeal taxa within the order *Thermoplasmatales* and class *Thermoplasmata*. Its exploration may also further expand the list of highly ranked taxa previously unknown to accommodate acidophilic members.

## Methods

### Sample collection and DNA extraction

Samples were collected from the surface layer (1–3 cm depth) of the water-saturated sediments of an acidic stream located in Parys Mt (North Wales, UK, 53° 23′ 13.6′′ 4° 20′ 58.6′′ W*)* in October, 2014. These samples were used for (1) bulk native DNA extraction for the metagenomic sequencing and SSU rRNA gene (full-length) PCR amplification (sample designated as ‘PM Mona’), and (2) for setting up enrichment cultures and consequent DNA extraction from these enrichments. Additional sampling was conducted at the same site in October 2016 (water and sediment as separate fractions taken from closely located spots) for barcoded amplicon sequencing. Water samples were filtered through Millipore Sterivex GV 0.2 μm syringe filters to recover the microbial biomass.

For enrichment cultures, the modified Medium DSMZ 88 (https://www.dsmz.de/microorganisms/medium/pdf/DSMZ_Medium88.pdf) was used [9]. The media was supplemented with Bacto™ beef extract, tryptone and casamino acids (BD Biosciences, Wokingham, UK), with each compound at a final concentration of 1 g l^−1^. For enrichment cultures, samples taken in October 2014 underwent three different treatments. Brief (5 s) vortexing and centrifugation at a low speed (10 min at 250 RCF) with subsequent (i) use of the pellet as the inoculum (‘enrichment from pellet’, EP), and (ii) collecting and using the supernatant produced above as the inoculum (‘enrichment from supernatant’, ES). Finally, (iii) the supernatant fraction (ES), which passed through the Millipore Sterivex GV 0.45 μm syringe membrane, was used as the inoculum for another enrichment culture (‘enrichment from filtrate’, EF).

The DNA from all samples was extracted using a MoBio PowerSoil® DNA Isolation Kit (Qiagen, Germany).

### Chemical analysis

The sediment samples were sieved to pass 2 mm and the stones (> 2 mm) retained, weighed and their volume calculated using a density value of 2.65 g cm^−3^. The water content of the sediment was determined by oven drying (105 °C, 24 h). Water- and air-filled porosity were calculated according to [49]. Total C and N were analysed with a Truspec® CN analyser (Leco Corp., St Joseph, MI). Metals in the samples were analysed using a S2 PicoFox TXRF Spectrometer (Bruker AXS Inc., Madison, WI). Electrical conductivity and pH were measured with standard electrodes. Soil redox potential was determined using a SenTix® probe (WTW Wissenschaftlich-Technische Werkstätten GmbH, Weilheim, Germany). The water samples were evaporated to dryness and the particulate fraction recovered and analysed as described above.

### Shotgun sequencing and analysis of Parys Mt metagenome

Shotgun metagenomic libraries were prepared from (i) environmental DNA extracted from samples taken in October 2014 (see Sample collection and DNA extraction), and (ii) genomic DNA extracted from enrichment cultures EF, ES and EP. DNA was fragmented using a Bioruptor sonicator (Diagenode, Belgium) to achieve an average fragment length of 500–700 bp. Further steps of library preparation were accomplished using NebNext® Ultra™ DNA Library prep kit for Illumina® according to the manufacturer’s instructions. Sequencing was performed on Illumina MiSeq™ platform using 500-cycles reagent kit. Then, 18.27, 1.88, 4.18 and 8.38 millions of read pairs were obtained for samples PM Mona, EP, ES and EF, respectively.

Quality trimming and filtering of reads was performed with CLC Genomics Workbench 9.5 (Qiagen, Germany) while overlapping reads were merged with the SeqPrep tool (https://github.com/jstjohn/SeqPrep/). Metagenomic assembly was performed using SPAdes (v. 3.10) without a read error correction step [50]. The resulting contigs were used only for the extraction of sequences of phylogenetic markers.

Community composition analysis of environmental metagenome (sample PM.Mona) was performed using raw sequencing reads, graftM package ver 0.11.1 [51] provided rpsB reference database, extended with sequences of *Cuniculiplasma divulgatum* PM4 (GCA_900090055.1), *Thermoplasmatales* archaea ‘A-plasma’ (GCA_000447225.1), ‘E-plasma’ (GCA_000496135.1), ‘I-plasma’ (GCA_001856825.1), ‘*Candidatus* Methanoplasma termitum’ (GCA_000800805.1), ‘*Ca*. Micrarchaeum’ sp. AZ1 (GCA_001896515.1), ‘*Ca*. Mancarchaeum acidiphilum’ (GCA_002214165.1), ‘*Ca*. Parvarchaeum acidiphilum’ ARMAN-4 (GCA_002412065.1), ‘*Ca*. Parvarchaeum acidophilus’ ARMAN-5 (GCA_002412085.1) as well as rpsB sequences from metagenomic bins related to TMEG-group and ‘*Ca*. Micrarchaeota’. Due to the distinct phylogenetic position of members of Parys Mt extremely acidophilic microbial community, and therefore the high possibility of wrong taxonomic affiliation, the reference database was extended by rpsB sequences, identified in our *shotgun* metagenomic assembly. All resulting sequencing were aligned and used to build an HMM model for the search step of graftM.

Sequences obtained from less diverse communities of enrichment cultures were analysed by CLARK k-mer-based sequence classification system [52]. In the first step, 16S and 23S were extracted using barrnap tool (https://github.com/tseemann/barrnap). All sequences were then aligned using blastn algorithm against refseq genomes, wgs and HTGS NCBI databases. The nearest genomic contigs were used as references for the creation of CLARK database. In the second step, preliminary metagenomic contigs, assembled with SPAdes, were classified with CLARK against NCBI-derived reference database (kmer = 32 was used for better reliability). After that step, CLARK reference database was complemented by reliably classified contigs. Finally, resulting database was used for the final classification of shotgun metagenomic reads (kmer = 22 was used for better reliability).

### 16S rRNA V3-V4 amplicon sequencing

Metabarcoding libraries of 16S rRNA amplicons corresponding to V3-V4 variable region were prepared by single PCR with double-indexed fusion primers as described by [53]. 16S rRNA annealing part of forward primers corresponded to Pro341F (CCTACGGGNBGCASCAG) primer described in [54]; reverse primers corresponded to modified R806 prokaryotic primer (GGACTACHVGGGTWTCTAAT) [55]. PCR amplification of 16S rRNA genes was performed by qPCRmix-HS™ SYBR mastermix (Evrogen, Russia) using the following conditions: 30 cycles of denaturation at 95 °C for 15 s; primer annealing at 58 °C, 15 s; DNA synthesis at 72 °C, 25 s, followed by final incubation for 5 min at 72 °C. Purification of PCR products was done using the Cleanup Mini kit (Evrogen, Russia). The quality of the final libraries was assessed using the electrophoresis in agarose gel.

Libraries were sequenced with MiSeq™ Personal Sequencing System technology of Illumina Inc. (San Diego, CA, USA) using paired-end 250-bp reads. Demultiplexing was performed as described previously [53]. After demultiplexing, all reads were subjected to stringent quality filtering, and parts of reads, corresponding to 16S rRNA primers were removed using CLC Genomics Workbench 10.0 (Qiagen, Germany). At the end, 18–22 thousand of reads were used for each analysed sample.

The resulting demultiplexed paired sequenced files for each sample were used as an input for the DADA2 high-resolution 16S amplicon analysis package [56] according to the standard pipeline published on DADA2 web page (https://benjjneb.github.io/dada2/index.html). As a result, 384 individual amplified sequenced variants (ASVs) were produced, while only 123 ASVs were represented by 10 or more reads in all samples analysed. Taxonomic classification of resulting ASVs was performed by Bayesian classification algorithm [57] built in DADA2 package against the Silva132 database [58]. Visualisation of data and calculation of alpha diversity metrics was performed using the R phyloseq package [59].

### 16S rRNA phylogenetic analysis

SSU rRNA gene sequence phylogenetic analysis for TMEG group was performed as described previously [9]. Briefly, a set of 16S rRNA nucleotide sequences assigned in the literature as related to TMEG-group were selected and aligned using Muscle algorithm [60]. Phylogenetic analysis was performed in MEGA6 Program [61] using the Maximum Likelihood method with 1000 repetitions bootstraps [62]. The general time-reversible model (GTR, G + I, four categories) [63] was used for inferring the evolutionary distances. The trees are drawn to scale, with branch lengths measured in the number of substitutions per site with corrections, associated with the model. All positions with less than 80% site coverage were eliminated. There were a total of 82 nucleotide sequences and 747 positions, involved in the calculations. The tree was displayed in packages ape [64] and phangorn [65] in the R programing environment.

Phylogenetic analysis for SSU rRNA genes sequences from barcode libraries and that extracted from the metagenomic sequences were performed using ARB-parsimony tool [66]. Briefly, all 16S rRNA gene sequences of V3-V4 fragments as well as that extracted from all four metagenomes (PM.Mona, EP, ES and EF) were added to the Silva SSU 132 Ref NR 99 arb file (including an alignment and a tree, https://www.arb-silva.de/download/arb-files/) where they have been co-aligned and placed on the Silva SSU 132 Ref NR 99 tree using the ARB-parsimony tool [66].

## Additional files

Additional file 1: **Figure S1.** Photos of sampling sites (A) and (B) and scheme of water flow in copper-containing sulfidic deposits (C). **Figure S2.** Shannon index for the sediment and water microbial communities. **Figure S3.** Rarefaction plot and species richness calculated in GraftM from the taxonomic annotation of the Parys Mountain metagenome sequencing data. **Table S1.** Soil pore water chemistry for the sediment and for the overlying surface water for the acidic stream of Parys Mt. (DOCX 860 kb)

Additional file 2: Metadata on TMEG-related sequences, used for diversity analysis of TMEG-related environmental clones. (XLS 40 kb)
