# Supplementary material for: Archaea dominate the microbial community in an ecosystem with low-to-moderate temperature and extreme acidity
Source: Microbiome. 2019 Jan 28;7:11. doi: 10.1186/s40168-019-0623-8 (PMC6350386; doi:10.1186/s40168-019-0623-8)
Supplement: Supplementary file 1 — Figure S1. Photos of sampling sites (A) and (B) and scheme of water flow in copper-containing sulfidic deposits (C). Figure S2. Shannon index for the sediment and water microbial communities. Figure S3. Rarefaction plot and species richness calculated in GraftM from the taxonomic annotation of the Parys Mountain metagenome sequencing data. Table S1. Soil pore water chemistry for the sediment and for the overlying surface water for the acidic stream of Parys Mt. (DOCX 860 kb) [file 40168_2019_623_MOESM1_ESM.docx]

**SUPPLEMENTARY INFORMATION**

**Archaea dominate the microbial community in an ecosystem with low-to-moderate temperature and extreme acidity**

**Aleksei A. Korzhenkov, Stepan V. Toshchakov, Rafael Bargiela, Huw Gibbard, Manuel Ferrer, Alina V. Teplyuk, David L. Jones, Ilya V. Kublanov, Peter N. Golyshin and Olga V. Golyshina**

**Figure S1 (A-C).** The pit pond (A) and mine water flow (B) sites are separated by approximately 7-8 m of copper-containing sulfidic deposits (C). The acidic water from the pond (pH 1.74, Eh +581 mV) flows through these deposits and emerges as surface water flow. Samples were taken from the water (SW) and sediment (SS) of the stream.


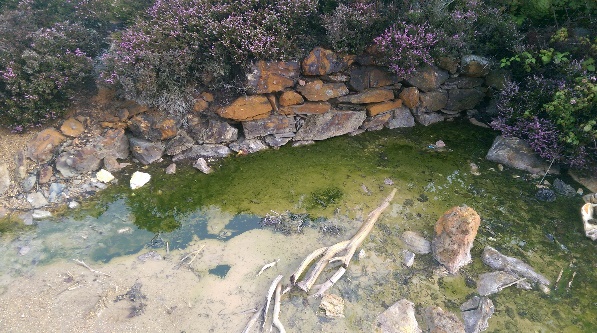


Pit pond

Mine water flow

= Copper containing sulfidic deposits


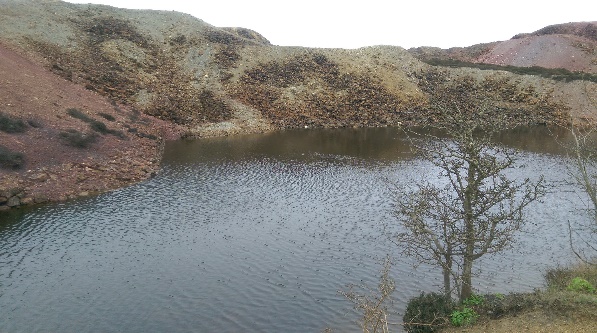


C.

A.

B.

LW

SW

SS


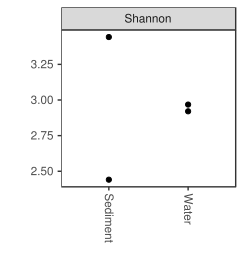


**Figure S2.** **Shannon index for the sediment and water microbial communities**,

calculated with Phyloseq package (McMurdie & Holmes, 2013).


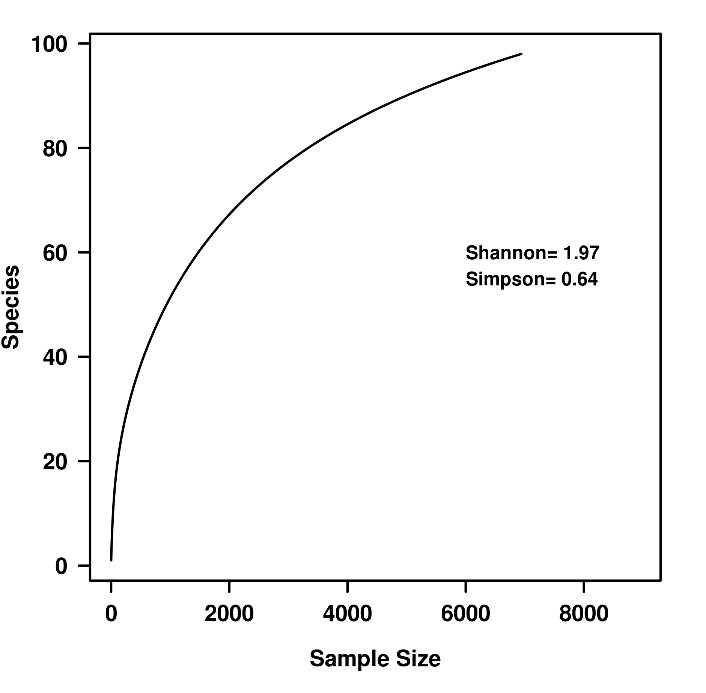


**Figure S3. Rarefaction plot and species richness calculated in GraftM from the taxonomic annotation of the Parys Mountain metagenome sequencing data.** Alpha-diversity Shannon and Simpson’s indices are shown in the figure. Both, rarefaction and diversity indexes were calculated under R environment using *vegan* package.

**Table S1. Soil pore water chemistry for the sediment and for the overlying surface water for the acidic stream of Parys Mt.** All values are expressed in mg l^-1^ and represent means ± SEM (*n* = 3).

|  | Surface water | |  | Sediment pore water | |
| --- | --- | --- | --- | --- | --- |
| Ca | 100.9 | ± 1.2 |  | 105.4 | ± 3.7 |
| Na | 37.0 | ± 0.2 |  | 99.8 | ± 14.0 |
| K | 4.9 | ± 0.3 |  | 44.0 | ± 41.2 |
| Mg | 93.0 | ± 18.6 |  | 121.1 | ± 6.3 |
| Fe | 1328 | ± 1 |  | 1863 | ± 36 |
| Cu | 90.4 | ± 0.2 |  | 49.7 | ± 2.4 |
| Zn | 94.7 | ± 0.1 |  | 105.7 | ± 6.7 |
| Pb | 5.7 | ± 0.1 |  | 12.1 | ± 0.5 |
| Ni | 0.24 | ± 0.00 |  | 0.43 | ± 0.05 |

**References**

McMurdie PJ, Holmes S. phyloseq: an R package for reproducible interactive analysis and graphics of microbiome census data. PLoS One*.*2013*;*8(4):e61217.
